# Ratio of Deaths from Chloroform

**Published:** 1867-11

**Authors:** 


*Ratio of Deaths from Chloroform.*—Dr. Andrews writes to the *Chicago Medical Examiner* an account of the mortality in the London Hospitals from the use of chloroform, in which he corrects several alleged misstatements in Sansom's recent valuable contribution to the literature of Anæsthesia. The question is so important and so well treated by Dr. Andrews that we copy the greater portion of his letter. It must be borne in mind that these statistics are purely Surgical.

London, July 13, 1867.

The London surgeons, with the exception of Dr. Protheroe Smith, and part of the officers of Guy's Hospital, have, with one consent, settled themselves down in the comfortable delusion that the risk of chloroform is scarcely worth considering. I find them just like Americans in one thing —they hate statistics. When I ask them what the actual risk of chloroformization is, they reply, "Oh, a very trifle, a mere nothing." The celebrated Mr. Simon, of Guy's Hospital, told me that it was in his opinion safer to take chloroform than to ride on a railroad car. Others, when pressed for an opinion, generally fall back on Mr. Sansom's Handbook on Chloroform, which estimates the deaths from Chloroform to be only one out of every 17,000 persons taking it. This may be set down as the opinion of the mass of London surgeons, both eminent and otherwise. Now, while they are gliding along in this agreeable state of mind, I have been at work to gather facts, and I find that instead of one death in 17,000, one patient dies out of every 3,461 anesthetized, in the very hospitals where these gentleman are at work. In other words, the deaths from chloroform under their own hands are about five times as numerous as they state, and as Mr. Sansom's Handbook estimates. If there were a railroad in London which killed one out of every 3,461 of its passengers I imagine one would hesitate to buy a ticket on it. In examining Mr. Sansom's statistics, to see how he arrives at his surprising estimate, I find the reason of his error. He omits entirely the London hospitals, and takes no note of the fact that several deaths from chlorororm occurred in the very one of which he was an officer. He obtains from some source an estimate of the number of times the article had been given in the hospitals of Birmingham, and other inland cities, amounting in all to 17,000 times. In these hospitals only one death from its use had "been reported," *ergo* chloroform causes only one death in 17,000 cases. Now, the very first step which I made in this investigation showed me that many deaths from anesthetics occur both in this country and America, which are never publicly reported, There are no regular statistics of anesthesia kept in any hospital here, that I have yet found, and as a chloroform death is an ugly uncomfortable fact, it usually slumbers unnoticed by the records. The difficulties of my investigation, therefore, have been very great. My mode of obtaining the facts had been this. I have made careful personal inquiries of the surgeons, house surgeons, dressers, secretaries of every hospital which I have visited, as to the two points, viz.: the number of times perannum chloroform has been given, as far back as their means of information extend, and, secondly, the number of deaths it has occasioned in the same period. These officers usually have no difficulty in estimating approximately the number of administrations per annum which have occured for several years, and the deaths during the same periods. In this way I have collected figures from fourteen hospitals in Liverpool and London. I obtained reliable accounts of chloroform being adminstered eighty-three thousand and fifty-nine times, (83,059,) and of these, twenty-four (24) proved fatal, or one in three thousand four hundred and sixty-one, (3,461.) Now if this is "safer than riding on a railroad," then I shall buy no more railroad tickets. A railroad in active business which should have a mortality like this would kill from 500 to 3,000 passengers every year, I shall continue my investigations on this subject both here and in France, and will report any new facts which I may ascertain Some of the hospitals here are a little uneasy, after all, and are using a new inhaler for safety, called Clover's apparatus, the principle of which is to inhale from a large air sack, which is inflated by a bellows, of a known capacity. The air, in passing from the bellows, goes through a hot evaporator, containing just enough chloroform by measure to give 3 or 3½ per cent. of chloroform vapor to the air in the sack. The huge, black bag, about 3 feet long and 2½ feet wlde, is slung on the back of an assistant, looking like the burden in the pictures on the back of Bunyan's Pilgrim. A large tube passes under his arm, terminated by an inhaler so arranged with valves that the patient inspires from the bag and expires into the open air. It is too clumsy for private practice, but works well in hospital, and probably promotes safety, as the patient cannot possibly get more than the per cent. of chloroform vapor which is placed in the sac. It has not been used enough yet to test, practically, the per cent. of mortality under it.

